# Eocene metatherians from Anatolia illuminate the assembly of an island fauna during Deep Time

**DOI:** 10.1371/journal.pone.0206181

**Published:** 2018-11-14

**Authors:** Grégoire Métais, Pauline M. Coster, John R. Kappelman, Alexis Licht, Faruk Ocakoğlu, Michael H. Taylor, K. Christopher Beard

**Affiliations:** 1 Centre de Recherche sur la Paléobiodiversité et les Paléoenvironnements, UMR 7207 (CNRS, MNHN, UPMC), Sorbonne Université, Muséum National d’Histoire Naturelle, Paris, France; 2 Biodiversity Institute, University of Kansas, Lawrence, Kansas, United States of America; 3 Department of Anthropology, University of Texas, Austin, Texas, United States of America; 4 Department of Earth and Space Sciences, University of Washington, Seattle, Washington, United States of America; 5 Department of Geological Engineering, Eskişehir Osmangazi University, Eskişehir, Turkey; 6 Department of Geology, University of Kansas, Lawrence, Kansas, United States of America; 7 Department of Ecology & Evolutionary Biology, University of Kansas, Lawrence, Kansas, United States of America; Institute of Vertebrate Paleontology and Paleoanthropology Chinese Academy of Sciences, CHINA

## Abstract

Island biotas have disproportionately influenced the history and development of evolutionary biology, but understanding their genesis and evolution across geological timescales has been hindered by a poor fossil record. Here we augment the insular Eocene (~43 Ma) mammalian fauna known from the Pontide terrane of central Anatolia by describing two new metatherian taxa (stem marsupials) from the Lülük Member of the Uzunçarşıdere Formation in the Orhaniye Basin. Geological and paleontological data indicate that the Pontide terrane was an island on the northern margin of Neotethys during the middle Eocene. Reflecting its geodynamic context in a region of active tectonic convergence, the Eocene Pontide terrane hosted a unique combination of Laurasian and Gondwanan mammals, including an anachronistic radiation of pleuraspidotheriids (archaic ungulates) that went extinct on the European mainland ~13 Ma earlier. Most of the mammalian clades occupying the Pontide terrane colonized it by dispersal across marine barriers rather than being stranded there through vicariance. Endemic radiations of pleuraspidotheriid ungulates and polydolopimorphian metatherians on the Pontide terrane reveal that *in situ* diversification was an important factor contributing to faunal assembly and evolution. The insular fauna that arose on the Pontide terrane is highly analogous to that of modern Sulawesi, which evolved under strikingly similar geological conditions. Illustrating the ephemeral nature of insular biotas across macroevolutionary timescales, the demise of the Pontide fauna coincided with paleogeographic changes enabling more cosmopolitan taxa to reach it for the first time. The high level of endemism shown by the mammalian fauna of the Uzunçarşıdere Formation eliminates the Pontide terrane as a potential early Eocene dispersal corridor between western Europe and India.

## Introduction

Island biotas have figured prominently in the development and elaboration of evolutionary biology [1, 2]. Documenting the processes related to faunal assembly in island contexts is therefore key to understanding broader patterns of dispersal, vicariance, adaptive radiation and extinction. While the recovery of Quaternary vertebrates in island contexts is relatively common [3], these data are typically more useful for illuminating geologically recent extinctions than the assembly and diversification of the island biotas being sampled. Significantly older fossil vertebrates from island contexts are comparatively rare [4–8], limiting the capacity of the fossil record to inform us about the genesis and evolution of island faunas across macroevolutionary timescales. As a result, it has been difficult to assess the extent to which tectonics and other geodynamic factors have influenced the assembly and evolution of island biotas and whether these complex interactions conform to predictable patterns [2].

**Fig 1:**
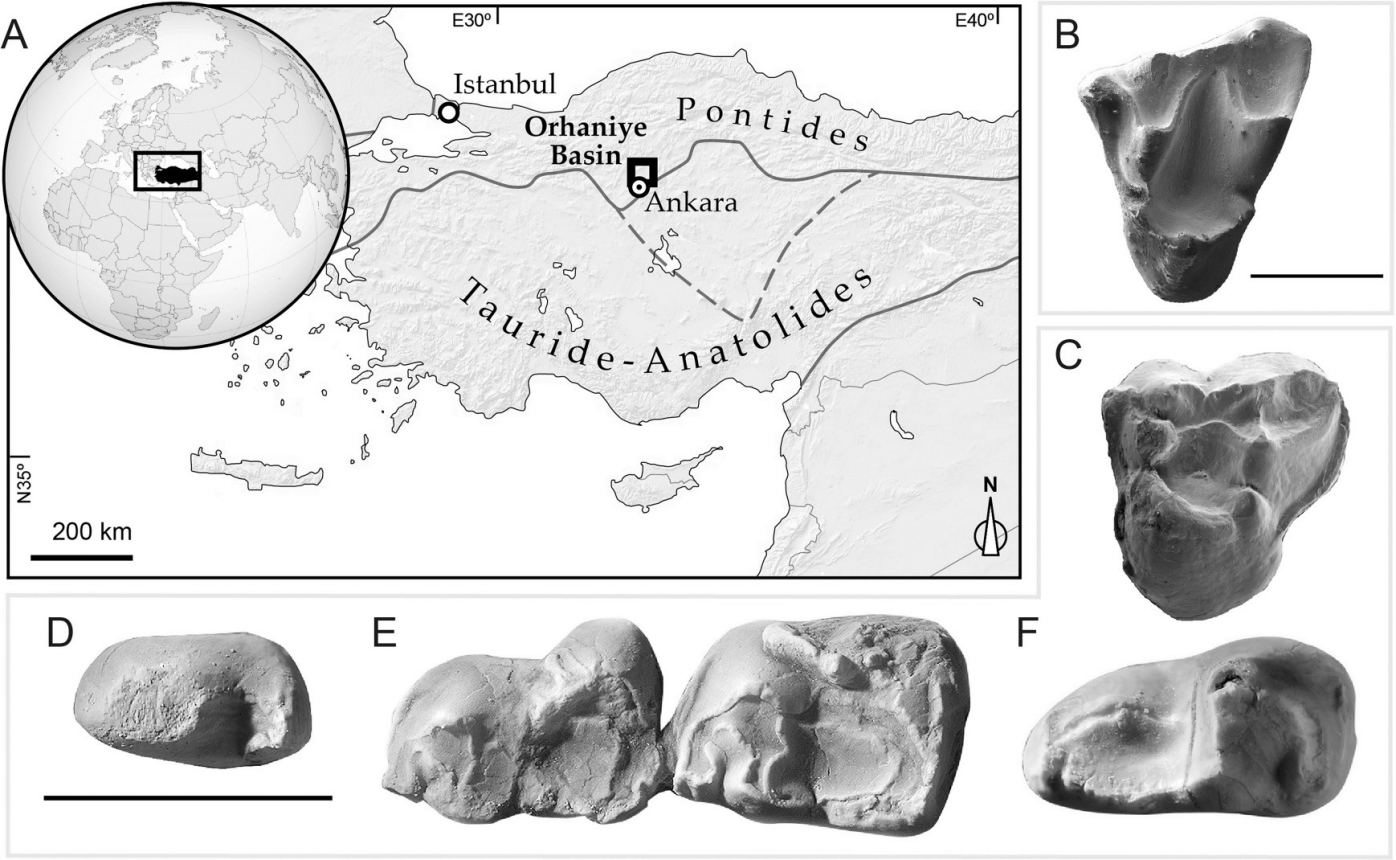
Location map and fossil metatherians from the Eocene Pontide terrane in central Anatolia. **A**, Map showing location of the Orhaniye Basin on the Pontide terrane as well as the location of major Anatolian suture zones. **B**, EOU-UCF-2, holotype left M^2^ in occlusal view of the herpetotheriid metatherian *Galatiadelphys minor*. Scale bar, 1 mm. **C**, EOU-UCF-3, left M^3^ in occlusal view of the anatoliadelphyid metatherian *Orhaniyeia nauta*. **D-F**, occlusal views of selected teeth preserved in EOU-UCF-4, holotype associated left and right dentary fragments of *Orhaniyeia nauta*. **D**, right P_3_; **E**, right M_1-2_; **F**, left M_4_. Scale bar for teeth of *Orhaniyeia nauta*, 5 mm.

Here we provide data bearing on the assembly of an endemic island fauna from the Orhaniye Basin on the Pontide terrane in what is now north-central Turkey (Fig 1). During the early Paleogene the Pontide terrane was part of an archipelago of microcontinents and volcanic arcs associated with the northward subduction of Tethyan oceanic lithosphere beneath the Eurasian plate [8]. A convergent plate margin, abundant volcanism, and significant changes in global eustasy would each have altered the dynamic paleogeography of this region throughout the early Paleogene. Eocene sedimentation in the Orhaniye Basin resulted from deformation, uplift and denudation associated with collisional tectonics between the Pontide terrane and the Kirşehir Massif and Tauride-Anatolide continent to the south. A persistent Paratethys Sea lay to the north and east of the Pontide terrane, while the Neotethys Sea separated it from Africa/Arabia to the south [8, 9]. The Lutetian mammal fauna known from the Lülük Member of the Uzunçarşıdere Formation in the Orhaniye Basin inhabited a fluviolacustrine ecosystem characterized by warm and wet, yet seasonally dry, climatic conditions [8]. Further details regarding the local tectonic setting, stratigraphy, geochronology, paleoclimate and paleoenvironment of the Uzunçarşıdere Formation are provided by Licht et al. [8].

Previous research on the middle Eocene Lülük Member of the Uzunçarşıdere Formation in the Orhaniye Basin has highlighted the uniquely endemic character of its mammalian fauna, which dates to ~43 Ma [8, 10–15]. In addition to the two new metatherians described below, mammalian taxa documented previously from this rock unit include one or more embrithopods [11], which are extinct large-bodied herbivores that are otherwise known primarily from the Afro-Arabian Plate [16, 17], four or five species of the endemic pleuraspidotheriid archaic ungulate *Hilalia* [12, 13], the relatively large-bodied metatherian *Anatoliadelphys maasae* [14], the palaeochiropterygid bat *Anatolianycteris insularis* [15], and fragmentary fossils of small insectivorous mammals that remain taxonomically enigmatic [11]. Surprising is the apparent absence of multiple placental mammal clades—including Rodentia, Carnivora, Creodonta, Perissodactyla and Artiodactyla—that are otherwise ubiquitous across Eurasia by the middle Eocene. The absence of these ecologically dominant placental clades and the low diversity and unbalanced character of the Eocene Pontide mammal fauna support its inferred island context.

The new metatherians described here are the first relatively small terrestrial mammals from the Eocene Pontide terrane to be diagnostic at suitably low systematic levels. Metatherians were broadly distributed and taxonomically diverse during the early Cenozoic, with many taxa showing restricted geographic and temporal ranges [18–21]. Accordingly, they provide an excellent window for reconstructing the chronology and likely processes controlling faunal assembly on the Pontide terrane.

## Materials and methods

### Specimens

The fossil metatherians reported here were collected from sites in the late middle Eocene Lülük Member of the Uzunçarşıdere Formation (UCF) in the Orhaniye Basin, north-central Turkey (Fig 1). Specimens were collected under the authority of a signed protocol between Eskişehir Osmangazi University and the University of Kansas. No permits were required, which complied with all relevant regulations. The permanent repository for the fossil specimens is Eskişehir Osmangazi University (EOU) in Eskişehir, Turkey, where they are publicly accessible. High-resolution epoxy casts of the fossils are maintained in the paleontological collections housed at the Muséum National d’Histoire Naturelle in Paris, France, and the Biodiversity Institute of the University of Kansas in Lawrence, Kansas, USA. Detailed data regarding the provenance of the individual fossils described here, including the type localities for newly described taxa, are maintained at Eskişehir Osmangazi University, the Muséum National d’Histoire Naturelle, and the Biodiversity Institute, University of Kansas.

### Nomenclatural acts

The electronic edition of this article conforms to the requirements of the amended International Code of Zoological Nomenclature, and hence the new names contained herein are available under that Code from the electronic edition of this article. This published work and the nomenclatural acts it contains have been registered in ZooBank, the online registration system for the ICZN. The ZooBank LSIDs (Life Science Identifiers) can be resolved and the associated information viewed through any standard web browser by appending the LSID to the prefix “http://zoobank.org/”. The LSID for this publication is: urn:lsid:zoobank.org:pub: 5B17C7A6-6E35-4BE1-94CF-4C7CFBD92D10. The electronic edition of this work was published in a journal with an ISSN, and has been archived and is available from the following digital repositories: PubMed Central, LOCKSS.

### Dental measurements and terminology

Standard dental measurements were obtained using digital Mitutoyo micrometers paired with a measuring stage under a Unitron Z6 binocular microscope equipped with an ocular reticle (Table 1). Dental terminology follows the nomenclature of Goin et al. [21]. Additionally, we follow Maga and Beck’s [14] usage of the term “exodaenodonty” to refer to cheek teeth in which the buccal side of the crown is distended farther basally than the lingual side of the crown.

**Table 1:** Dental measurements for new metatherians from the middle Eocene of Turkey.

Specimen/Taxon	Position	Locality	Mesiodistal length	Buccolingual width
***Galatiadelphys minor***				
EOU-UCF-2 (holotype)	left M^2^	Locality AK-01A	1.61	1.81
***Orhaniyeia nauta***				
EOU-UCF-3	left M^3^	ORH type Loc.	4.03	4.38
EOU-UCF-4 (holotype)	right P_3_	ORH type Loc.	4.15	2.40
EOU-UCF-4 (holotype)	right M_1_	ORH type Loc.	4.72	3.19
EOU-UCF-4 (holotype)	right M_2_	ORH type Loc.	4.79	3.61
EOU-UCF-4 (holotype)	left M_2_	ORH type Loc.	4.74	3.54
EOU-UCF-4 (holotype)	left M_4_	ORH type Loc.	5.74	3.14
EOU-UCF-4 (holotype)	right M_4_	ORH type Loc.	5.69	3.29
EOU-UCF-5	right M_4_	*Anatoliadelphys* type Loc.	6.10	3.46
EOU-UCF-6	left M_3_	Flat Top Loc.	4.59	3.39

All measurements are in mm.

### Phylogenetic analysis

Phylogenetic analyses were based on a data matrix (Table 2) that augments one first compiled by Chornogubsky and Goin [22]. Chornogubsky and Goin’s matrix consists of 45 morphological characters and 26 taxa. We added four taxa (*Palangania brandmayri*, *Orhaniyeia nauta*, *Anatoliadelphys maasae*, and *Thylacotinga bartholomaii*) and the following six characters to the data matrix published by Chornogubsky and Goin [22]:

46. StB on upper molars: conical (0), crested (1).
47. Paraconule on upper molars: large (0), small (1), absent (2).
48. Reduced trigon on upper molars: absent (0), present (1).
49. Relative size of StB and StD on upper molars: StB>StD (0), StB<StD (1).
50. Progressive development of exodaenodonty from M_1_-M_4_: absent (0), present (1).
51. Hypertrophy of protoconid on M_4_: absent (0), present (1).

The final matrix includes 30 taxa and 51 characters.

**Table 2:** Character-taxon matrix used in phylogenetic analyses.

*Alphadon*	?	0	0	0	0	0	0	0	0	0	0	0	0	0	0	0	0	0	0	0	0	0	0	0	0	0	0	0	0	0	0	0	0	0	0	0	0	0	0	0	0	0	-	0	0	0	0	0	0	0	1
*Pucadelphys*	0	0	0	0	0	0	0	0	0	0	0	0	0	1	0	0	0	0	0	0	0	0	0	0	0	0	0	0	0	0	0	0	1	0	1	0	1	0	0	0	0	0	?	0	0	0	0	0	0	0	1
*Derorhynchus*	?	1	0	2	0	0	?	0	0	?	?	?	?	?	0	0	0	0	0	0	0	0	0	0	0	0	0	0	0	0	0	0	2	0	1	0	1	0	0	0	0	0	?	0	0	0	1	0	0	0	0
*Dracolestes*	?	?	?	?	?	?	?	?	0	?	?	?	?	?	1	?	1	1	0	0	0	?	?	?	?	?	?	0	?	0	0	0	2	2	1	0	1	0	0	0	0	?	?	0	?	1	2	0		0	0
*Riolestes*	?	?	?	?	?	?	?	?	0	1	0	0	1	0	1	0	0	1	0	0	0	?	?	?	?	?	?	0	?	0	0	?	?	?	?	?	?	?	?	?	?	?	?	?	?	1	2	0	?	0	0
*Stilotherium*	?	0	1	1	0	0	0	1	0	0	1	0	1	1	1	1	1	1	1	0	0	1	0	0	1	0	1	0	0	0	0	0	3	2	1	0	2	0	1	0	0	2	0	0	0	1	2	0	?	0	0
*Rhyncholestes*	1	0	1	1	0	0	0	1	0	2	1	0	1	1	1	1	1	1	1	1	0	1	0	0	1	0	1	0	1	0	0	0	3	2	1	0	2	0	1	0	0	2	0	0	-	1	2	0	?	0	0
*Caenolestes*	1	0	1	1	0	0	0	1	0	2	1	0	1	1	1	1	1	1	1	1	0	1	0	0	1	0	1	0	1	0	0	0	3	2	1	0	2	0	1	0	0	2	0	0	-	1	2	0	?	0	0
*Pliolestes*	?	1	1	1	2	?	?	1	0	2	1	0	1	1	1	1	1	1	1	1	0	1	1	0	1	0	1	0	0	0	0	?	?	?	?	?	?	?	?	?	?	?	?	?	?	1	2	0	?	0	0
*Pichipilus*	?	0	1	1	0	0	0	1	0	2	1	0	0	1	1	1	0	1	0	0	1	1	1	0	0	0	1	1	0	1	1	0	3	2	1	0	2	0	1	0	0	2	0	0	0	1	2	0	?	0	0
*Phonocdromus*	?	0	1	1	0	?	?	1	0	2	1	0	0	1	1	1	0	1	0	0	1	1	1	0	0	0	1	1	0	1	1	0	3	2	1	0	2	0	1	0	0	2	0	0	0	1	2	0	?	0	0
*Palaeothentes*	0	1	2	1	0	0	0	1	1	1	1	0	0	2	0	2	0	1	0	0	1	2	1	1	0	0	1	1	0	0	?	0	?	2	1	0	2	0	1	0	0	2	0	0	0	1	2	0	?	0	0
*Acdestis*	0	1	2	1	2	0	1	1	1	1	1	0	0	2	0	2	0	1	0	0	1	2	1	1	0	0	1	1	0	0	0	0	?	2	1	0	2	0	1	0	0	2	0	0	0	1	2	0	?	0	0
*Abderites*	?	1	2	1	2	2	0	1	2	1	?	1	?	?	0	2	0	1	0	0	1	2	1	1	0	0	1	1	1	0	0	0	?	2	1	0	2	0	1	0	0	2	0	0	-	1	2	0	?	0	0
*Parabderites*	?	0	2	1	0	?	?	1	2	1	?	1	?	0	0	2	0	1	0	0	1	2	1	1	0	0	1	1	1	0	0	0	?	2	1	0	2	0	1	0	0	2	0	0	-	1	2	0	?	0	0
*Microbiotherium*	0	1	0	0	0	0	0	0	0	0	0	0	0	1	0	0	0	0	0	0	0	0	0	1	0	0	0	0	0	0	0	1	0	0	0	1	1	1	?	0	0	?	?	0	-	?	1	0	0	0	0
*Glasbius*	?	0	?	?	0	0	1	0	0	0	0	0	0	1	0	0	0	0	0	0	0	1	1	0	0	0	0	0	0	0	0	1	0	1	0	1	1	1	0	0	0	0	?	0	0	1	1	0	0	0	0
*Prepidolops*	?	1	?	?	1	2	1	0	0	0	1	0	0	1	0	0	0	0	0	0	0	1	0	0	0	1	0	0	1	0	0	1	4	1	0	1	1	1	0	0	0	1	?	0	0	?	2	0	0	0	0
*Bonapartherium*	0	1	?	?	1	2	1	0	0	1	?	0	?	1	0	0	0	0	0	0	0	1	0	1	0	1	0	0	1	0	0	1	4	1	0	1	3	1	0	0	0	1	1	0	0	?	2	0	0	0	0
*Proargyrolagus*	0	1	?	1	0	1	1	1	0	0	?	0	?	1	0	1	0	0	0	2	0	2	?	1	?	?	0	0	1	0	0	0	?	?	?	1	3	1	?	0	0	1	1	0	-	?	2	0	0	0	0
*Klohnia*	?	1	3	1	0	1	1	1	0	0	?	0	?	1	0	?	?	0	?	2	0	2	?	1	2	?	0	0	1	0	0	1	4	0	0	0	3	1	0	0	0	1	1	0	0	?	2	0	0	0	0
*Roberthoffstetteria*	?	0	?	?	0	?	?	?	0	0	0	0	1	1	0	0	0	0	0	0	0	1	0	1	0	1	0	0	1	0	0	1	0	0	0	2	4	1	0	1	1	0	0	1	0	?	1	0	0	0	0
*Sillustania*	?	?	?	?	?	?	?	?	0	?	?	?	?	?	?	?	?	?	?	?	?	?	?	?	?	?	?	0	?	?	?	1	0	0	0	2	3	1	0	1	1	0	1	1	0	?	1	0	0	0	0
*Polydolops*	?	1	3	1	1	0	1	1	0	2	?	0	?	?	0	3	0	0	0	0	0	2	0	1	2	1	0	1	1	0	0	1	5	1	1	1	4	1	0	1	1	0	0	1	-	0	2	0	0	0	0
*Epidolops*	0	1	2	2	1	2	1	0	1	1	1	0	0	0	0	3	0	0	0	0	0	2	1	1	1	1	0	0	1	0	0	1	4	1	0	1	3	1	0	0	0	1	1	0	0	?	2	0	0	0	0
*Palangania*	?	?	?	?	?	?	?	?	0	0	0	0	0	0	0	0	0	0	0	2	0	0	0	1	?	0	?	0	0	0	?	1	1	1	0	0	1	1	0	1	0	0	?	0	0	0	1	1	0	?	?
*Chulpasia*	?	?	?	?	?	?	?	?	0	0	0	0	0	0	0	0	0	0	0	0	0	0	0	0	?	?	?	0	0	0	0	1	1	1	0	0	1	1	0	1	0	0	?	0	0	0	1	1	0	?	1
*Orhaniyeia*	?	?	?	?	?	?	?	?	0	0	0	0	0	0	0	0	0	0	0	2	0	0	0	0	?	0	?	0	0	0	0	1	1	1	0	0	1	1	0	1	0	1	?	0	0	1	2	1	1	1	1
*Anatoliadelphys*	?	1	?	?	0	0	1	0	0	0	0	0	0	1	0	0	0	0	0	0	0	0	0	0	?	0	?	0	0	0	0	1	1	1	0	0	-	1	0	1	0	1	?	0	0	1	2	1	1	1	1
*Thylacotinga*	?	?	?	?	?	?	?	?	0	0	0	0	0	0	0	0	0	0	0	0	0	0	0	0	?	0	?	0	0	0	0	1	1	1	0	0	1	1	0	1	0	1	?	0	0	1	1	0	0	1	0

Maximum parsimony analyses of this updated character-taxon matrix were performed with TNT [23] using a Tree Bisection Reconnection search with 1000 replicates and 10 trees saved by replication. Among the new characters, only the multistate character 47 was treated as ‘ordered’ or ‘additive’ because it corresponds to a natural morphocline. The remaining characters were treated as in [22]. TNT recovered 55 trees with a best score of 146 steps, with a consistency index (CI) of 0.50 and a retention index (RI) of 0.79. A standard bootstrap analysis was performed using 10,000 replicates with the ‘fast’ stepwise addition option.

## Results

### Systematic palaeontology

Mammalia Linnaeus, 1758
Metatheria Huxley, 1880
Herpetotheriidae Trouessart, 1879

*Galatiadelphys* gen. nov. urn:lsid:zoobank.org:act:D7EB2454-8841-44D2-B60A-5D914E65706C.

*Galatiadelphys minor* sp. nov. urn:lsid:zoobank.org:act:F40F881B-0B9F-4459-A0E0-67AC3E8BB7CE.

#### Etymology

“Galatia” was a name for the Ankara region of Anatolia during antiquity. Greek “delphys” (womb) is a commonly used suffix in metatherian systematics. Latin “minor” (less) refers to the small size of this species.

#### Holotype

**Fig 2:**
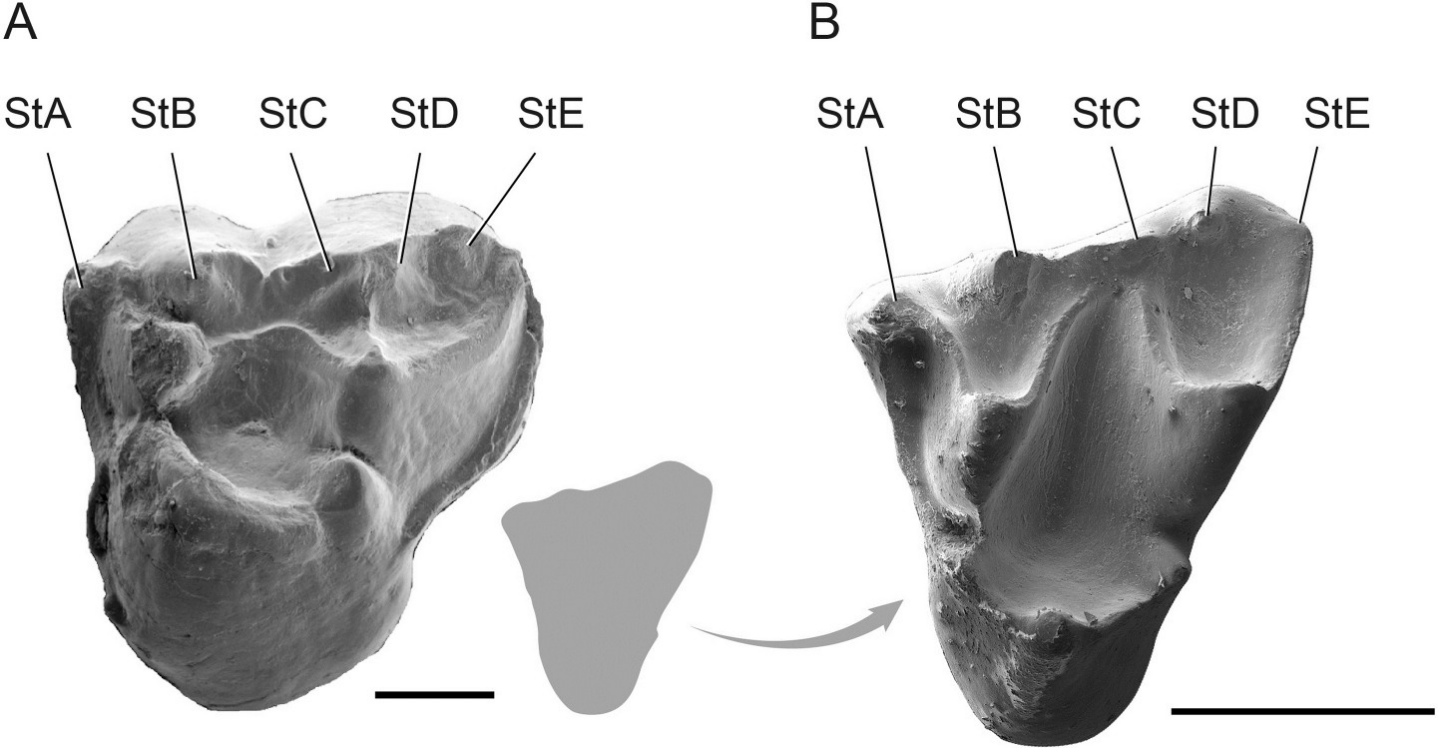
Upper molars of metatherians from the Eocene of Anatolia scaled to have the same anteroposterior length. **A**, EOU-UCF-3, left M^3^ of *Orhaniyeia nauta*. **B**, EOU-UCF-2, holotype left M^2^ of *Galatiadelphys minor*. Actual size of *Galatiadelphys minor* relative to *Orhaniyeia nauta* is shown by the gray outline of *Galatiadelphys minor* (located between the two images). Stylar cusps are labelled. Scale bars equal 1 mm.

EOU-UCF-2, left M^2^ (Figs 1B and 2B).

#### Hypodigm

In addition to the holotype; AK95-34, right upper molar fragment; AK95-35, right M^4^; and AK94-8, left lower molar. These additional specimens of *Galatiadelphys minor* were figured by Kappelman et al. [10].

#### Locality and age

Locality AK-01A, late middle Eocene, Lülük Member of the Uzunçarşıdere Formation, Orhaniye Basin, Turkey.

#### Diagnosis

Differs from *Peratherium* and other herpetotheriids in having strongly dilambdodont upper molars with a buccolingually narrow stylar shelf, relatively straight buccal margin, and conules that are situated near the lingual bases of paracone and metacone.

#### Description

The holotype upper molar (anteroposterior length: 1.61 mm; buccolingual width: 1.81 mm) is triangular in outline with three main cusps, two small conules, and five stylar cusps of unequal size. The protocone is voluminous and connected to the paraconule and metaconule by sharp crests, the posprotocrista being longer than the preprotocrista. The conules are pyramidal in shape, similar in size, and each is situated near the base of the corresponding buccal cusp (paracone and metacone). The metacone is larger and more voluminous than the paracone, which is anteroposteriorly compressed and delimited anteriorly by a strong anterior cingulum. The buccal margin of the stylar shelf is nearly straight, whereas the ectoflexus is typically present and shallow in the vicinity of StC in *Peratherium* and other herpetotheriids [19]. The postparacrista and premetacrista are directed buccally, where they independently reach the base of StC. Consequently, there is a considerable buccal expansion of the trigon basin. The preparacrista arcs anteriorly to join StA, which forms the buccal border of the anterior cingulum. StC takes the form of a low spur (rather than being cuspate), and StE is barely discernible. StB is the largest and most prominent stylar cusp, followed in size by StA and StD, respectively. StE is barely visible, being almost completely fused with the buccal extremity of the postmetacrista. The buccal surfaces of the paracone and metacone are strongly excavated and buccally concave, which is exaggerated by the prominent development of dilambdodonty in this taxon. Overall, the breadth of the stylar shelf is less than what is typical in herpetotheriids.

The remaining teeth in the hypodigm of *Galatiadelphys minor* were thoroughly described by Kappelman et al. [10]. Here, we note that these specimens are appropriate in size and morphology to pertain to the same species as the holotype. In particular, the upper molars AK95-34 and AK95-35, which were tentatively identified as right M^3^ and right M^4^ [10], resemble the holotype of *Galatiadelphys minor* in terms of their dilambdodonty and relatively straight buccal margins.

#### Comparisons

The most striking features of EOU-UCF-2 are the buccal orientation of the postparacrista and premetacrista (which contributes to the strong dilambdodonty evident in this taxon), the narrow breadth of the stylar shelf, and the robustness of the protocone. Another purported metatherian showing strong development of dilambdodonty is *Garatherium mahboubii* from the early Eocene of El Kohol, Algeria [24]. *Garatherium mahboubii* is based on a unique upper molar (possibly M^3^) that is about 30% smaller than the holotype of *Galatiadelphys minor*, which is regarded here as M^2^. In *Garatherium mahboubii*, and in contrast to EOU-UCF-2, the buccally directed postparacrista and premetacrista merge before reaching the columnar StC. The holotype of *Garatherium mahboubii* also differs from that of *Galatiadelphys minor* in showing continuity between the preparacrista and StB, having a shallow and symmetrical ectoflexus, having a reduced anterior cingulum, and having a slightly “waisted” occlusal outline caused by a constriction between the protocone and the buccal cusps as well as having an anteroposteriorly shorter and buccolingually wider protocone. Although *Garatherium mahboubii* was initially described as a peradectine metatherian [24], recent workers have been unanimous in regarding it as a eutherian mammal. Gheerbrant [25] was the first to suggest that *Garatherium mahboubii* is a dilambdodont member of the Adapisoriculidae, an extinct clade of eutherian mammals known from the early Cenozoic of Europe and Africa and the Late Cretaceous of India [26, 27]. The phylogenetic position of Adapisoriculidae is debated. Recent hypotheses have reconstructed adapisoriculids either as stem eutherians [26] or as crown placentals showing affinities with either euarchontans [28, 29] or afrosoricids [30]. Reassessing the affinities of *Garatherium mahboubii* is beyond the scope of this paper, but we see no reason to postulate a special relationship between the latter taxon and *Galatiadelphys minor*.

Although the upper molars of herpetotheriids such as *Peratherium*, *Herpetotherium* and *Asiadidelphis* typically bear a centrocrista that is somewhat deflected buccally [19, 31, 32], none of these taxa shows the fully formed dilambdodonty that characterizes *Galatiadelphys minor*. However, with the exception of this feature and the concomitant reduction of the stylar shelf, EOU-UCF-2 most closely resembles European herpetotheriids, particularly early Eocene species of the genus *Peratherium*, such as *P*. *constans* from Dormaal, Belgium [19]. Beyond being strongly dilambdodont, EOU-UCF-2 differs from upper molars of *P*. *constans* in having a StB that does not coalesce with the parastyle, in lacking an ectoflexus, and in having a protocone that is less asymmetrical anteroposteriorly. Another feature that distinguishes the Turkish specimen is the narrow breadth of its stylar shelf, although this character varies somewhat according to tooth locus in *P*. *constans* and other herpetotheriids.

Anatoliadelphyidae fam. nov. urn:lsid:zoobank.org:act:A1EB4FFC-BC6F-4F1C-B859-F2D35933D2A1

#### Type genus

*Anatoliadelphys* Maga and Beck, 2017.

#### Included genera

The type genus and *Orhaniyeia* gen. nov.

#### Diagnosis

Differs from *Palangania*, Chulpasiinae, and all other metatherians in having the following combination of features: bunodont lower molars with weakly cuspidate entoconids becoming progressively exodaenodont posteriorly; lingual margin of lower molar talonids lined with multiple small neomorphic cuspules; trigonid of M_4_ with hypertrophied protoconid; upper molars with narrow stylar shelves on which StB and StD are enlarged and closely approximated with paracone and metacone, respectively; stylar cusps tend to be connected by anteroposteriorly oriented crests; upper molar protocones hypertrophied and asymmetrical, with enlarged posterolingual face; M^3^ strongly asymmetrical because of posterobuccal expansion of postmetacrista and related structures.

*Orhaniyeia* gen. nov. urn:lsid:zoobank.org:act:4B11B943-0618-4A46-809A-6414D18BD416

*Orhaniyeia nauta* sp. nov. urn:lsid:zoobank.org:act:FAD3AAFB-58CB-4A7E-A4EF-8D2DE1A0DC23

#### Etymology

Generic name reflects the geographic provenance of this taxon. Latin “nauta” (sailor) alludes to the inferred mode of dispersal employed by ancestral Anatoliadelphyidae in reaching the Pontide terrane.

#### Holotype

**Fig 3:**
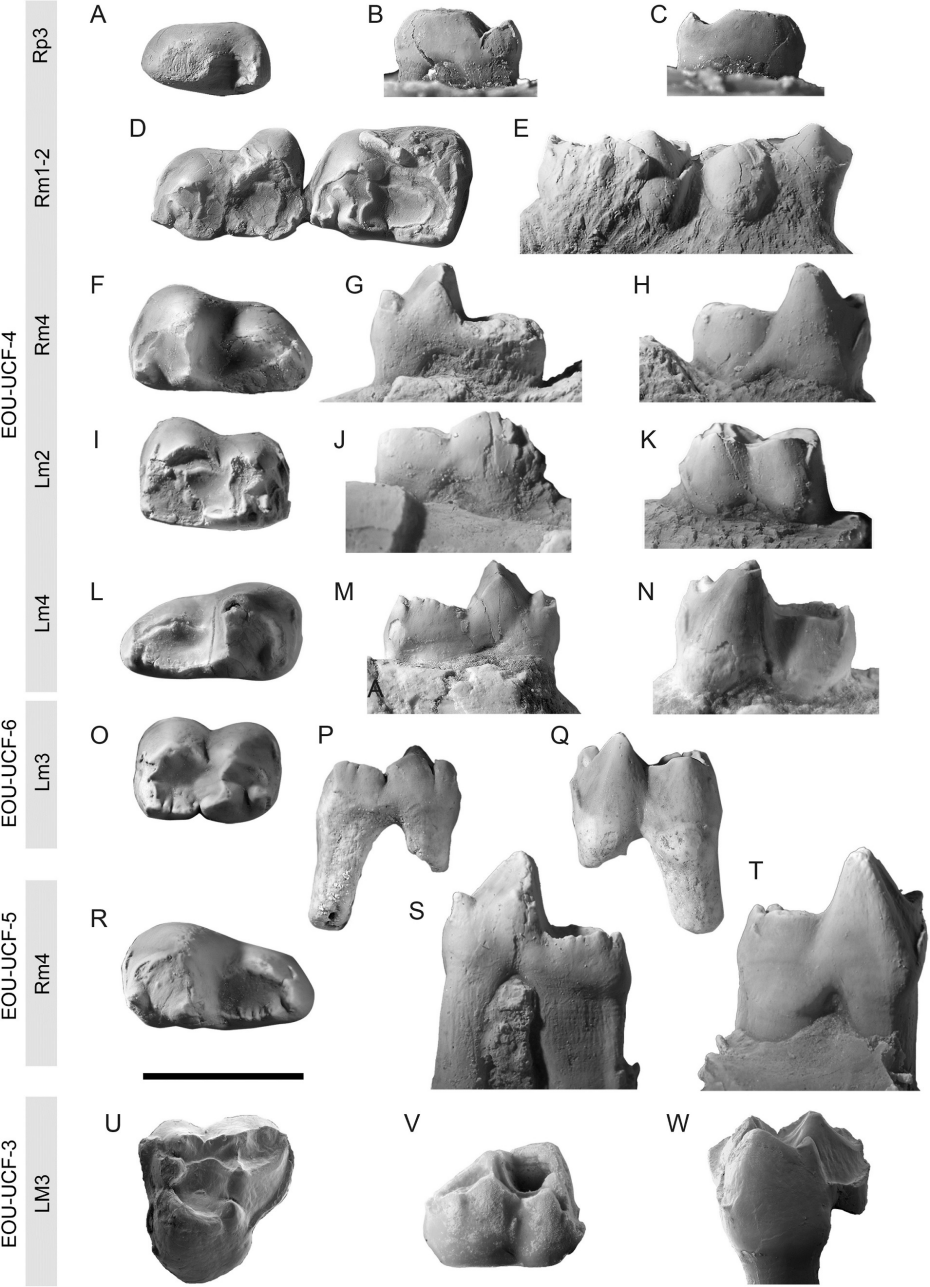
Dental remains attributed to *Orhaniyeia nauta*. Specimen numbers are shown to the left. **A-C**, right P_3_ included as part of holotype in occlusal (**A**), lingual (**B**), and buccal (**C**) views. **D-E**, right dentary fragment preserving M_1-2_ included as part of holotype in occlusal (**D**) and buccal (**E**) views. **F-H**, right dentary fragment preserving M_4_ included as part of holotype in occlusal (**F**), lingual (**G**), and buccal (**H**) views. **I-K**, left dentary fragment preserving M_2_ included as part of holotype in occlusal (**I**), lingual (**J**), and buccal (**K**) views. **L-N**, left dentary fragment preserving M_4_ included as part of holotype in occlusal (**L**), lingual (**M**), and buccal (**N**) views. O-Q, left M_3_ in occlusal (**O**), lingual (**P**), and buccal (**Q**) views. **R-T**, right dentary fragment preserving M_4_ in occlusal (**R**), lingual (**S**), and buccal (**T**) views. U-W, left M^3^ in occlusal (**U**), buccal (**V**), and lingual (**W**) views. Scale bar equals 5 mm.

EOU-UCF-4, associated dentary fragments and teeth preserving left M_2_ and M_4_ and right P_3_, M_1-2_, and M_4_ (Figs 1D–1F and 3A–3N).

#### Hypodigm

In addition to the holotype; EOU-UCF-3, left M^3^ (Figs 1C, 2A and 3U–3W); EOU-UCF-5, right dentary fragment preserving M_4_ (Fig 3R–3T); EOU-UCF-6, left M_3_ (Fig 3O–3Q); and AK95-19, left M^1^ or M^2^. The latter specimen was figured by Maas et al. [11].

#### Locality and age

ORH type locality, late middle Eocene, Lülük Member of the Uzunçarşıdere Formation, Orhaniye Basin, Turkey.

#### Diagnosis

P_3_ differs from that of *Anatoliadelphys* in being narrower relative to crown length, retaining a distinct talonid heel, and being less hypertrophied in relation to lower molars; lower molars differ from those of *Anatoliadelphys* in being much less exodaenodont and in lacking the marked progressive size increase posteriorly found in that genus. M^3^ differs from that of *Anatoliadelphys* in having a continuous centrocrista, and in retaining a stronger metaconule, distinctly cuspate StB, and small StA and StC.

#### Description

EOU-UCF-3 is relatively unworn and is tentatively identified as M^3^ due to the asymmetry caused by its posterobuccal expansion. The asymmetrical outline of the molar is accentuated by the anterior displacement of the protocone, which is almost aligned transversely with the paracone and StB, and the posterobuccal expansion of the postmetacrista. The protocone is by far the most voluminous and prominent cusp. Reflecting the anterior position of the protocone, the preprotocrista is oriented primarily buccally to join the base of the paracone, while the postprotocrista is oriented almost directly posteriorly, joining the base of the metaconule. Both pre- and postprotocristae are trenchant, and the latter structure is continuous with a high lingual wall that slopes steeply toward the base of the crown (Fig 3W). The trigon is deep, buccolingually narrow and nearly circular in outline. The metacone is taller than the paracone, which is anteroposteriorly compressed. There is no paraconule, whereas a prominent and columnar metaconule is closely appressed to the base of the metacone. The preparacrista is faint and joins the base of the weak StA, whereas the trenchant postmetacrista is long and prominent, extending to the posterobuccal corner of the tooth. The continuous centrocrista arcs buccally, forming an obtuse angle that is concave lingually. The stylar shelf is reduced in breadth, and the stylar cusps bear sharp crests that are predominantly oriented anteroposteriorly. StB and StD are situated close to the paracone and metacone, respectively, effectively being twinned with them. StD is the largest and most prominent stylar cusp, whereas StC is not particularly distinct, forming a tiny bulge on the blade-like anterior crest of StD. There is a distinct StE, although the posterobuccal corner of the tooth is slightly damaged. A transverse crest links StD to the metacone; this crest is absent between StB and the paracone, being replaced by a deep groove extending anteroposteriorly. The anterior cingulum is weak, although it extends from StA to the level of the protocone lingually. The posterior cingulum is stronger, extending from the posterobuccal corner of the molar to the level of the metaconule. The enamel is relatively thick.

P_3_ in the holotype is double-rooted and subrectangular in occlusal outline. The crown is simply constructed, consisting of a trigonid that lacks either a paraconid or a metaconid and a short, narrow talonid heel. The protoconid is large and bulbous, occupying about two-thirds of the premolar’s length. Apical wear has obscured the unworn height of the protoconid, but it is clear that P_3_ was not particularly high-crowned. A weak crest bearing a noticeable wear facet descends the postvallid, where it becomes continuous with a second crest that is likely homologous with the cristid obliqua. The buccal side of the crown is inflated, such that there is no development of a hypoflexid. The talonid is longer than wide, and it bears a small cusp (possibly homologous with the hypoconid) near its posterior border. The posterolingual part of the talonid suffers from minor breakage, obscuring whether a small entoconid may have been present there. The ratio of anteroposterior length/buccolingual width for P_3_ is 1.86, while the equivalent ratio is 1.43 for *Anatoliadelphys maasae*, reflecting the greater degree of inflation of P_3_ in the latter taxon.

In contrast to the condition in *Anatoliadelphys maasae* [14], the lower molars do not increase markedly in size posteriorly in *Orhaniyeia nauta*. For example, in *Anatoliadelphys maasae* the anteroposterior length of M_4_ (9.8 mm) is roughly 188% of the anteroposterior length of M_1_ (5.2 mm). In contrast, in *Orhaniyeia nauta* the anteroposterior length of M_4_ is only 121% of the anteroposterior length of M_1_ (using EOU-UCF-4 as the basis for comparison) (Table 1). The lower molars of *Orhaniyeia nauta* are moderately exodaenodont; that is, the buccal side of each lower molar crown extends farther ventrally than does its lingual side (Fig 3). The degree of exodaenodonty shown by the lower molars of *Orhaniyeia nauta* appears to increase from front to back, as is also the case in *Anatoliadelphys maasae*. However, the progressive development of exodaenodonty from M_1_ to M_4_ shown by *Anatoliadelphys maasae* is much greater than what is seen in *Orhaniyeia nauta*, as is evident by comparing the highly exodaenodont M_4_ of *Anatoliadelphys maasae* [14] with the moderately exodaenodont M_4_ of *Orhaniyeia nauta* (Fig 3H, 3N and 3T). The lower molars of *Orhaniyeia nauta* are sub-rectangular in outline, and the talonids are buccolingually broader than the trigonids on all lower molars aside from M_4_. Lower molar trigonids and talonids are about equal in terms of anteroposterior length, and each trigonid projects above its corresponding talonid. The metaconid is more voluminous and taller than the paraconid on each lower molar. The protoconid is the tallest and most prominent lower molar cusp, which is especially well-marked on M_4_. Variably developed pre- and postcingulids are present on all lower molars. The entoconid and hypoconulid occur on the lingual border of the talonid, but neither of these cusps is conical or strongly cuspidate. Well preserved lower molars such as EOU-UCF-6 show that multiple neomorphic cuspules are present along the lingual margin of the lower molar talonids. A similar condition occurs in *Anatoliadelphys*. The hypoconid is more voluminous than the entoconid, which is displaced anteriorly. There is a distinct notch on the pre-entocristid (best observed in EOU-UCF-6; see Fig 3O and 3P), and the cristid obliqua terminates buccal to the midpoint of the protocristid. The postcristid extends transversely and ventrally and meets the buccal extension of the hypoconulid at approximately the midpoint of the posterior border of the talonid. The talonid of M_4_ is elongated and narrow. Due to the weakness of the metaconid, the protocristid of M_4_ is not V-shaped in posterior view as it is on M_1-3_.

#### Comparisons

In terms of its dental morphology, *Orhaniyeia nauta* most closely resembles *Anatoliadelphys maasae*, although the latter is about twice as large as the former. However, several distinctive features justify the generic distinction between these taxa, most of which indicate that *Anatoliadelphys* is autapomorphous with respect to *Orhaniyeia* and other closely related metatherians.

If our interpretation of EOU-UCF-3 as being an M^3^ is correct, M^3^ of *Orhaniyeia* differs from that of *Anatoliadelphys* in having a much lower-crowned posterobuccal margin (this region is strongly exodaenodont in *Anatoliadelphys*), having a continuous centrocrista, in retaining distinct (although reduced) StA and StC, in retaining more distinctly cuspate (rather than crestiform) StB, and in having a well-defined and relatively bulbous metaconule. M^3^ of *Orhaniyeia* bears an unusual (and presumably neomorphic) crest connecting StD and the metacone. Maga and Beck [14] interpret a similar crest on M^3^ of *Anatoliadelphys* as a buccally oriented and “distinctly notched” premetacrista. Because M^3^ of *Orhaniyeia* retains both the crest connecting StD and the metacone and the premetacrista (as part of its continuous centrocrista), we homologize the crests connecting StD and the metacone in these taxa. As a result, M^3^ of *Anatoliadelphys* apparently lacks a distinct premetacrista, yielding its discontinuous centrocrista.

The lower molars of *Anatoliadelphys maasae* are characterized by a progressive and rather marked increase in size from M_1_ to M_4_ [14], which does not occur in *Orhaniyeia nauta* (Table 1). However, the lower molars of *Orhaniyeia nauta* otherwise resemble those of *Anatoliadelphys maasae* in many ways. In both taxa the lower molars are bunodont and exodaenodont, although the degree of exodaenodonty shown by the posterior lower molars of *Anatoliadelphys* far exceeds that which is seen in *Orhaniyeia*. Likewise, in both taxa the talonids of M_1-3_ are broadly basined, while that of M_4_ is narrow and elongated. In both *Orhaniyeia* and *Anatoliadelphys* the entoconid and hypoconulid are closely associated on the posterolingual side of the lower molar talonids, and neither structure is strongly cuspidate or conical. Instead, the entoconid and hypoconulid are joined by additional neomorphic cuspules that line the lingual margin of the lower molar talonids. Lower molar trigonids, especially that of M_4_, are dominated by a hypertrophied protoconid.

Outside of the Anatoliadelphyidae, the dentition of *Orhaniyeia* compares best with that of phylogenetically basal, early Paleogene polydolopimorphians such as *Palangania brandmayri* from the early and/or middle Eocene of Patagonia [21, 33, 34] and, to a lesser extent, *Chulpasia mattaueri* from the late Paleocene or early Eocene of Peru [20, 35], and *Chulpasia jimthorselli* and *Thylacotinga bartholomaii* from the early Eocene of Australia [20].

In terms of its upper and lower molar morphology, *Orhaniyeia* most closely resembles *Palangania*. Notable among the many shared features are the buccolingually narrow stylar shelf, the buccal arc of the centrocrista, and the close approximation between StB and the paracone and StD and the metacone, respectively. It is worth noting that M^1^ on the holotype of *Palangania brandmayri* lacks StC, which is nonetheless present on M^2^, indicating that the presence of StC can vary along the tooth row. The upper molars of *Palangania brandmayri* differ from those of *Orhaniyeia nauta* in showing a more bulbous StA, having StB larger than StD, having weaker anteroposteriorly oriented crests linking the stylar cusps and lacking a posterior cingulum. The reduction of the trigon basin is somewhat less marked and the protocone is not as broad and prominent on upper molars of *Palangania brandmayri* as is the case in *Orhaniyeia nauta*. Tejedor et al. [34] reported a single upper molar (tentatively identified as M^3^) of *Palangania* sp. from the middle Eocene site of La Barda, Patagonia. This molar is significantly smaller (~40%) than those of *Palangania brandmayri*, and it further differs from the latter in possibly lacking a metaconule (obliterated by wear on the specimen) and retaining a paraconule. However, the La Barda upper molar of *Palangania* sp. resembles that of *Orhaniyeia nauta* in having a very reduced trigon and an anteriorly displaced and relatively asymmetrical protocone. The lower molars of *Orhaniyeia nauta* resemble those of *Palangania brandmayri* in being bunodont and moderately exodaenodont, with broad talonids showing similar anterior displacement of the entoconid (except for M_4_), and slight posterior displacement of the metaconid (except for M_4_). However, the lower molars of *Orhaniyeia nauta* differ from those of *Palangania brandmayri* in lacking a crest linking the metaconid and paraconid and having a more lingual position of the hypoconulid.

Aside from *Palangania*, *Orhaniyeia* most closely resembles the early Paleogene polydolopimorphians *Chulpasia* and *Thylacotinga* in terms of its upper and lower molar morphology. The latter two genera are thought to be closely related, and together they comprise the subfamily Chulpasiinae [20]. The upper molars of *Chulpasia* and *Thylacotinga* resemble those of *Orhaniyeia* in many ways, particularly in having relatively narrow stylar shelves with StB and StD enlarged and closely approximated with the paracone and metacone, respectively. However, the upper molar morphology of chulpasiines differs from that of *Orhaniyeia* in retaining a paraconule (at least in *Chulpasia*), lacking a posterobuccally expanded postmetacrista, having StB larger than StD, and retaining a buccolingually broader trigon and a more central position for the protocone. Lower molars of *Chulpasia mattaueri* have been described [35], which resemble those of *Orhaniyeia nauta* in being fairly bunodont (though less so than in *Palangania*) and moderately exodaenodont, in having lower molar trigonids in which the protoconid is substantially larger than either the paraconid or the metaconid, and in having anteriorly displaced entoconids. However, the lower molars of *Chulpasia mattaueri* differ from those of *Orhaniyeia nauta* in having more trenchant crests (particularly the paracristid, cristid obliqua and postcristid), retaining more distinctly cuspidate entoconid and hypoconulid, and having the hypoconulid more posterior and less lingual in position. The lower molars of *Thylacotinga* resemble those of *Orhaniyeia* in having a trigonid as long anteroposteriorly as the talonid, the orientation of the cristid obliqua, the lingual position of the hypconulid, and the presence of pre- and postcingulids. However, the lower molar morphology of *Thylacotinga* differs from that of *Orhaniyeia* in having a relatively larger metaconid (so that the disparity between protoconid and metaconid is less pronounced), in retaining a distinct notch between the hypoconulid and entoconid, and in having a larger entoconid that is less displaced anteriorly.

## Discussion

### Assembly and demise of the insular Pontide mammal fauna

The phylogenetic and biogeographic affinities of the metatherians and other mammals from the Eocene Pontide terrane illuminate how its geodynamic context—including ancient climatic conditions and fluctuating paleogeographic relationships with adjacent landmasses—strongly influenced the assembly, composition, and eventual demise of the insular fauna inhabiting this Tethyan archipelago.

**Fig 4:**
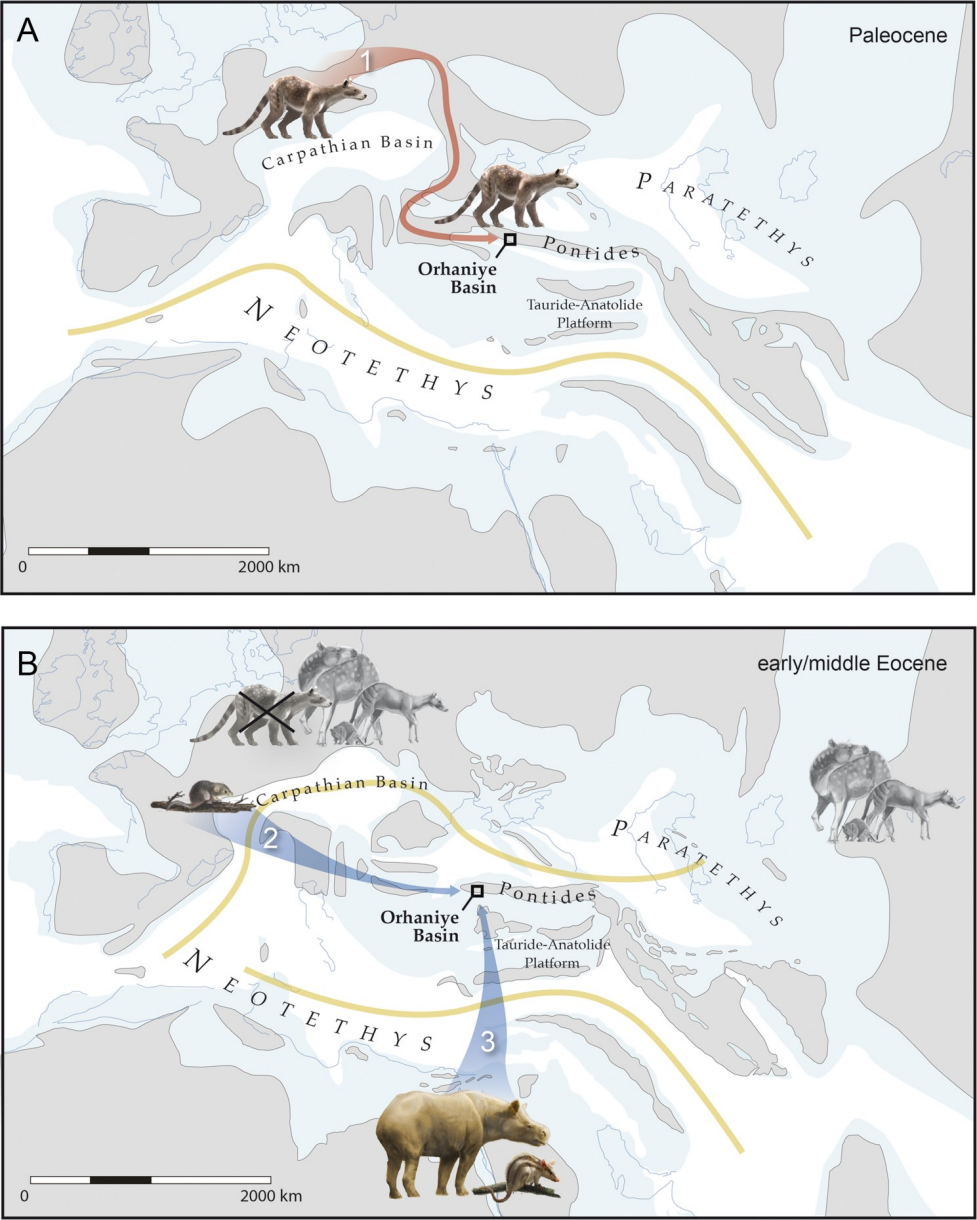
Assembly of the insular Eocene mammalian fauna occupying the Pontide terrane. Regional paleogeography is based on geological data [8, 9] and paleontological data discussed in the text. **A**, European Paleocene pleuraspidotheriid ungulates colonized the Pontide terrane, possibly by traversing ephemeral land bridges during intervals of low eustatic sea level (track 1). During the late Paleocene, terrestrial continuity between the Pontide terrane and Eurasia was severed by a combination of eustatic changes and tectonics, stranding pleuraspidotheriids on the Pontide terrane prior to the appearance of modern ungulates and rodents in adjacent parts of Eurasia. **B**, Modern ungulates and rodents become ubiquitous across Eurasia by the earliest Eocene, while pleuraspidotheriids are extirpated there. Multiple Eocene mammal clades, including the herpetotheriid ancestors of *Galatiadelphys* (track 2), embrithopods and the anatoliadelphyid ancestors of *Orhaniyeia* (track 3), disperse to the Pontide terrane by rafting or swimming across parts of Neotethys. Modern ungulates and rodents fail to colonize the Pontide terrane until it becomes reconnected to the Eurasian mainland later in the Paleogene.

The dominant mammalian herbivores on the Eocene Pontide terrane were an endemic radiation of pleuraspidotheriid archaic ungulates belonging to the genus *Hilalia* [12, 13]. The oldest documented occurrence of pleuraspidotheriids on the Pontide terrane dates to the late early Eocene, based on *Parabunodon anatolicum* from the Çeltek Formation in the Suluova Basin, about 350 km east of the Orhaniye Basin sites that yield *Hilalia* [36]. Aside from these Eocene Anatolian taxa, pleuraspidotheriids have only been reported from the Paleocene of western Europe, where they were locally abundant [37]. European pleuraspidotheriids became extinct at the Paleocene-Eocene boundary, coinciding with the local first appearance of modern mammalian ungulates including perissodactyls and artiodactyls. Based on their restricted geographic and stratigraphic ranges elsewhere, pleuraspidotheriids must have occupied the Pontide terrane during the Paleocene. An interval of lower eustatic sea level during the late Paleocene may have enabled pleuraspidotheriids to colonize the Pontide terrane by traversing ephemeral land bridges connecting it to adjacent parts of the European mainland (Fig 4A). However, whatever continuity existed between the Pontide terrane and adjacent parts of Eurasia must have been severed before the Paleocene-Eocene boundary, based on the absence of rodents, perissodactyls and artiodactyls in the middle Eocene sites of the Orhaniye Basin (Fig 4). The persistence and diversification of pleuraspidotheriids on the Pontide terrane for ~13 Ma after their extirpation on the European mainland was facilitated by this insular context, which freed them from competition with modern mammalian ungulate clades.

Following the isolation of the Pontide terrane prior to the Paleocene-Eocene boundary, any mammals that were not already in residence would have been forced to disperse across marine barriers to colonize this landmass. Likely examples include embrithopods and the metatherians described above. Embrithopods have a stratigraphic range on the Pontide terrane encompassing most of the Eocene [38]. Embrithopods are conventionally allied with Paenungulata (including modern proboscideans, hyracoids, sirenians, and their extinct relatives), which are themselves a major component of the placental mammal clade Afrotheria [16]. Assuming that their afrotherian affinities imply an African origin, embrithopods must have dispersed across Neotethys to colonize the Pontide terrane from the south (Fig 4B). Turkish embrithopods are commonly recovered from lignites representing swampy paleoenvironments, and the African embrithopod *Arsinoitherium* shows postcranial features that are consistent with a semiaquatic lifestyle [39]. Hence, embrithopods may have colonized the Pontide terrane by swimming and island hopping across Neotethys.

Both of the new Eocene metatherians described here are inferred to have colonized the Pontide terrane by rafting, but their divergent phylogenetic affinities suggest that their biogeographic origins must have been very different. The herpetotheriid *Galatiadelphys* belongs to a clade that is well-documented in western Europe, where they first appear at the beginning of the Eocene [19]. Herpetotheriids have occasionally been reported from later Paleogene sites in Asia and Africa [18, 32], but these are all substantially younger than *Galatiadelphys*. Given the marked temporal disparity documented for herpetotheriids across the Old World and the morphological similarities between *Galatiadelphys* and early European herpetotheriids such as *Peratherium constans*, *Galatiadelphys* probably colonized the Pontide terrane by rafting there from Europe (Fig 4B). Herpetotheriids could not have traversed hypothetical Paleocene land bridges connecting Europe with the Pontide terrane because their oldest European occurrence is earliest Eocene [19]. Based on criteria recently established with respect to early anthropoid primates [40], small-bodied and presumably arboreal herpetotheriids would have been excellent candidates for overwater dispersal by rafting, particularly during Eocene hyperthermal and/or monsoonal climatic episodes. Indeed, the ability of herpetotheriids to disperse by rafting across Tethys is already implied by their early Oligocene occurrence in Egypt, well before the tectonic collision between Afro-Arabia and Eurasia near the Oligocene-Miocene boundary [18].

**Fig 5:**
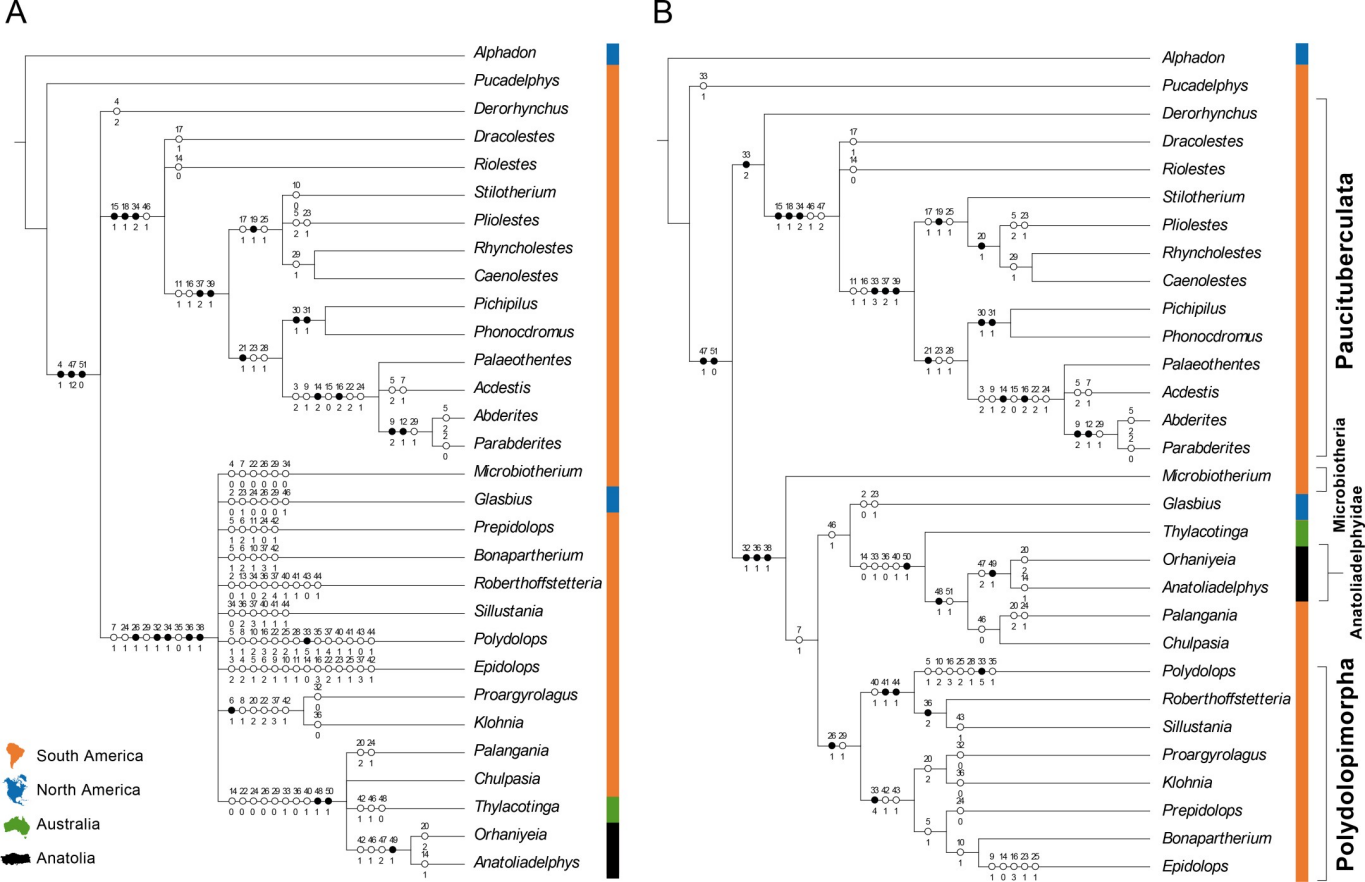
Results of maximum parsimony analyses of character-taxon matrix provided in Table 2. **A**, Strict consensus tree; **B**, 50% majority-rule consensus tree. Synapomorphies supporting each node are listed along each branch. For each node the list of the non-ambiguous synapomorphies is given, each synapomorphy being represented by a black circle (strict synapomorphy) or an open white circle (homoplasic synapomorphy). The upper numbers indicate the character number and the lower numbers indicating the states for these characters.

**Fig 6:**
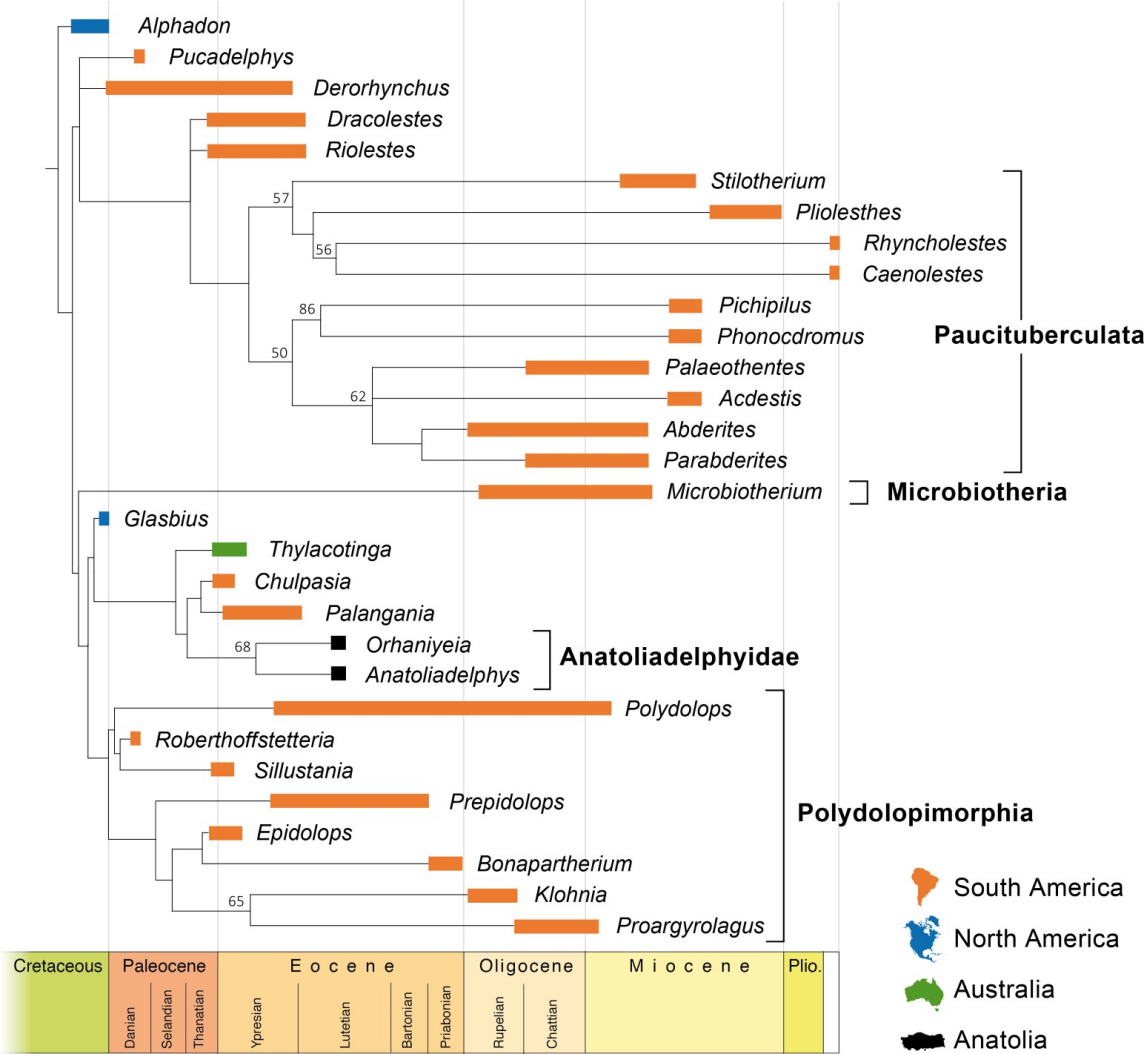
Phylogenetic position of endemic anatoliadelphyid metatherians from the Pontide terrane. Tree topology is based on 50% majority-rule consensus tree shown in Fig 5B. Temporal axis is shown at the bottom.

The endemic anatoliadelphyid metatherians *Orhaniyeia nauta* and *Anatoliadelphys maasae* [14] rank among the most unexpected components of the middle Eocene mammal fauna known from the Pontide terrane. Our phylogenetic analyses reconstruct *Orhaniyeia* and *Anatoliadelphys* as sister taxa that are closely related to South American and Australian bunodont polydolopimorphian metatherians such as *Palangania*, *Chulpasia* and *Thylacotinga* [20, 21] (Figs 5 and 6). Given their apparent phylogenetic ties with Gondwanan polydolopimorphians, reconstructing how ancestral anatoliadelphyids reached the Pontide terrane is debatable. The nearest major Gondwanan landmass to the Pontide terrane during the early Paleogene was Africa, but polydolopimorphians have never been reported from the African fossil record. However, the African fossil record is widely acknowledged to be inadequate, particularly during the early and middle Eocene when anatoliadelphyids most likely colonized the Pontide terrane [41]. We hypothesize that basal polydolopimorphians were actually present in Africa during the early and/or middle Eocene, whence they rafted northward across Neotethys to colonize the Pontide terrane (Fig 4B). Obviously, this hypothesis needs to be tested by future paleontological exploration in the early and middle Eocene of Africa.

The uniquely insular mammal fauna that arose on the Eocene Pontide terrane is highly analogous to that of modern Sulawesi [42], which evolved under similar circumstances in a different region of active tectonic convergence between major Laurasian and Gondwanan landmasses. In both cases, dispersal across marine barriers appears to have been far more important than vicariance in terms of faunal assembly. With the exception of the anachronistic pleuraspidotheriids, all major mammal clades known from the Eocene Pontide terrane are inferred to have colonized it by rafting, swimming or flying (Table 3). Among the modern mammals of Sulawesi, only the phalangerid marsupials seem to be old enough to have potentially arrived by vicariance [42]. In situ diversification occurred in both cases, exemplified by multiple species of *Hilalia*, embrithopods and anatoliadelphyids on the Pontide terrane and endemic radiations of tarsiers, macaques, shrews and cuscuses on Sulawesi. Further similarities between the Eocene fauna of the Pontide terrane and that of modern Sulawesi include the divergent (Laurasian and Gondwanan) origins of their constituent taxa and the dearth or absence of carnivorans and perissodactyls in both faunas (there are no native perissodactyls on Sulawesi and the viverrid *Macrogalidia* is its only native carnivoran). Despite important differences with respect to space, time and (hence) taxa, the numerous similarities shown by the assembly of the mammal faunas of modern Sulawesi and the Eocene Pontide terrane suggest that geological context is a crucial factor impacting the composition and diversity of island biotas [2].

**Table 3:** Summary of faunal assembly on the Pontide terrane.

Taxon	Inferred mode of emplacement	Geographic affinities	Evidence for *in situ* diversification?
*Galatiadelphys minor* (Herpetotheriidae)	Dispersal	Laurasia: Europe	No
*Orhaniyeia nauta* (Anatoliadelphyidae)	Dispersal	Gondwana: Africa	yes (sister group of *Anatoliadelphys maasae*)
*Anatoliadelphys maasae* (Anatoliadelphyidae)	Dispersal	Gondwana: Africa	yes (sister group of *Orhaniyeia nauta*)
*Hilalia saribeya* (Pleuraspidotheriidae)	Vicariance	Laurasia: Europe	yes (polytypic genus)
*Hilalia selanneae* (Pleuraspidotheriidae)	Vicariance	Laurasia: Europe	yes (polytypic genus)
*Hilalia sezerorum* (Pleuraspidotheriidae)	Vicariance	Laurasia: Europe	yes (polytypic genus)
*Hilalia robusta* (Pleuraspidotheriidae)	Vicariance	Laurasia: Europe	yes (polytypic genus)
*Hilalia* sp. nov. (Pleuraspidotheriidae)	Vicariance	Laurasia: Europe	yes (polytypic genus)
*Hypsamasia seni* (Palaeoamasiidae)	Dispersal	Gondwana: Africa	yes (sister group of *Palaeoamasia*)
*Palaeoamasia* sp. (Palaeoamasiidae)	Dispersal	Gondwana: Africa	yes (sister group of *Hypsamasia seni*)
*Anatolianycteris insularis* (Palaeochiropterygidae)	Dispersal	Laurasia: either Europe or Asia	No
Undescribed primate (Omomyidae)	Dispersal	Laurasia: Asia	No

The demise of the insular fauna that occupied the Pontide terrane almost certainly coincided with paleogeographic changes enabling more cosmopolitan taxa to colonize the Pontide terrane for the first time. The timing of this important episode of faunal turnover remains weakly constrained, but it occurred prior to the late Oligocene, when a cosmopolitan fauna dominated by Eurasian ruminants, rhinocerotoids, rodents, and other taxa is documented in the Çankiri-Çorum Basin of central Anatolia [43, 44]. Possibly, the endemic Eocene Pontide fauna was replaced earlier, but the persistence of embrithopods until near the Eocene-Oligocene boundary in the Boyabat Basin of northern Anatolia [38] suggests that the Pontide terrane remained insular until the end of the Eocene.

### Broader implications for old world paleobiogeography

Smith et al. [45] recently proposed that early Eocene terrestrial mammal faunas from India could have dispersed to or from western Europe across various islands lying along the northern margin of Neotethys, such as the Pontide terrane and the Kohistan-Ladakh island-arc system. This hypothetical dispersal pathway potentially explains the biogeographic affinities that have been proposed between the early Eocene Vastan faunas of India and contemporary faunas from western Europe. Although earliest Eocene mammal faunas remain unknown in Anatolia, the highly endemic middle Eocene fauna documented from the Pontide terrane indicates that this part of the northern margin of Neotethys did not function as a corridor for early Eocene terrestrial mammals dispersing between Europe and India. A more likely hypothesis explaining biogeographic patterns shown by terrestrial mammals from the early Eocene of western Europe and India is that Asia was the proximate source for the modern mammal clades found in both of the latter regions [46, 47], precluding any need for island-hopping across the northern margin of Neotethys during the early Eocene. The limited evidence that is currently available regarding fossil bats from the Pontide terrane [15] suggests that volant taxa may have been able to disperse along this pathway while terrestrial mammals were unable to do so.
