# Correction: Eocene metatherians from Anatolia illuminate the assembly of an island fauna during Deep Time

**DOI:** 10.1371/journal.pone.0212985

**Published:** 2019-02-22

**Authors:** Grégoire Métais, Pauline M. Coster, John W. Kappelman, Alexis Licht, Faruk Ocakoğlu, Michael H. Taylor, K. Christopher Beard

The third author’s name is spelled incorrectly. The correct name is: John W. Kappelman. The correct citation is: Métais G, Coster PM, Kappelman JW, Licht A, Ocakoğlu F, Taylor MH, et al. (2018) Eocene metatherians from Anatolia illuminate the assembly of an island fauna during Deep Time. PLoS ONE 13(11): e0206181. https://doi.org/10.1371/journal.pone.0206181

## References

[1] Métais G, Coster PM, Kappelman JR, Licht A, Ocakoğlu F, Taylor MH, et al (2018) Eocene metatherians from Anatolia illuminate the assembly of an island fauna during Deep Time. PLoS ONE 13(11): e0206181 10.1371/journal.pone.0206181 30427946 PMC6235269

